# Editorial: Specialized proresolving mediators: Benefits within and beyond inflammation resolution in cardiometabolic, neurological and psychiatric disorders

**DOI:** 10.3389/fphys.2023.1176700

**Published:** 2023-04-04

**Authors:** Teresa Sousa, Dora Pinho, Antonio Recchiuti, Elisa Isopi, Waldiceu A. Verri

**Affiliations:** ^1^ Departamento de Biomedicina—Unidade de Farmacologia e Terapêutica, Faculdade de Medicina, Universidade do Porto, Porto, Portugal; ^2^ Centro de Investigação Farmacológica e Inovação Medicamentosa (MedInUP), Universidade do Porto, Porto, Portugal; ^3^ Department of Medical, Oral and Biotechnological Science (DSMOB), Università degli Studi “G. D'Annunzio” Chieti-Pescara, Chieti, Italy; ^4^ Department of Pathology, Londrina State University, Londrina, PR, Brazil

**Keywords:** specialized proresolving lipid mediators, inflammation, cardiometabolic disorders, neurological diseases, psychiatric disease

The acute inflammatory process is a protective and ideally self-limited response aimed at neutralizing infectious, traumatic, ischemic, toxic and autoimmune aggressions. However, uncontrolled inflammation can be profoundly deleterious. Indeed, in the last two decades, enormous paradigm shifts have occurred with the recognition of inflammation as a central pathophysiological mechanism of several prevalent and harmful diseases and, more recently, with the uncovering of an active resolution of inflammation response mediated by a large family of specialized proresolving mediators (SPM), such as lipoxins, resolvins, protectins and maresins. Since then, new avenues have been opened for the understanding and treatment of a broad range of central and peripheral pathologies, from neurological to cardiovascular disorders. This Research Topic aimed to gather the current knowledge and shed light on the pharmacological role and therapeutic potential of endogenous and exogenous SPM in neurological and cardiovascular diseases, focusing not only on their proresolving and anti-inflammatory effects, but also on their pleiotropic impact. Five papers, including two reviews and three original research articles, were published within this Research Topic.

Starting by the review articles, Li et al. focused on the beneficial effects of resolvins in neurological diseases. They first presented a general overview of the endogenous biosynthesis of resolvins and their intracellular signaling pathways, with more specific detail on resolvin receptors and cellular targets in the central nervous system. In the second part, the authors provided evidence from clinical and preclinical studies on the protective role of resolvins in neurodegenerative (Alzheimer’s disease, Parkinson’s disease, amyotrophic lateral sclerosis), neuroimmune (multiple sclerosis, Guillain-Barré syndrome) and cerebrovascular disorders (ischemic stroke), ending up with a discussion about their therapeutic potential in neurological disorders and current limitations to their clinical application. A second review paper of Zaninelli et al. approached the pathophysiological role of SPM in arthritis. Authors extensively described SPM classes and presented a structured overview of the current knowledge, according to the precursor lipid substrate: arachidonic acid, eicosapentaenoic acid (EPA) and docosapentaenoic/docosahexaenoic acid (DPA/DHA). Both preclinical and clinical data supporting the contribution of SPM to disease status and potential therapeutic use in arthritis were reviewed.

Regarding the original papers, there were two neurological-related studies addressing the analgesic effects of SPM and one cardiovascular research article investigating the relationship between glucocorticoid exposure, lipoxin A_4_ (LXA_4_) dampening and preeclampsia. The paper by Tao et al. demonstrated the analgesic effect of 1 kHz spinal cord stimulation in an established (2–3 weeks post-induction) rat model of spared nerve injury-induced neuropathic pain. Quite remarkably, authors described decreased central and peripheral levels of the pro-inflammatory mediator IL-1β, and increased central production of the SPM resolvin D1 (RvD1), which support a fundamental role of modulation of neuroinflammation in the analgesic effects of spinal cord stimulation, in neuropathic pain context. Further evidence supporting this hypothesis was provided by an additional experiment reported in the paper, where intrathecal RvD1 administration produced an analgesic pattern similar to that obtained with spinal cord stimulation. Zhao et al. showed the analgesic effect of intrathecal protectin DX (PDX) in a lumbar radicular pain rat model, induced by non-compressive lumbar disc herniation. According to these authors, PDX treatment reverts the pro-/anti-inflammatory imbalance associated with the model, acting through facilitation of the spinal cord autophagy flux, and activation of adenosine monophosphate-activated protein kinase signaling. Lastly, Liu et al. paralleled the study of pregnant women samples and of a rat model of preeclampsia to demonstrate that the induction of this disease by glucocorticoids depends on the reduction of endogenous LXA_4_ production, thus placing LXA_4_ insufficiency as a disease mechanism in preeclampsia. Noteworthy, LXA_4_ exerted several protective pleiotropic effects (reduction of placental oxidative stress, amelioration of intrauterine growth restriction, antagonism of glucocorticoid effects on placental 11β-HSD2 expression and trophoblast development) in experimental preeclampsia, thus showing a potential for the prevention or treatment of this severe gestational disease associated with multisystemic inflammation and endothelial dysfunction.

Summing up, pre-clinical and clinical data presented in the articles of this Research Topic align to support not only the importance of SPM in inflammation resolution, but also their usefulness as pharmacological approaches to treat neurological and cardiovascular diseases, including neurodegenerative, neuroimmune and cerebrovascular disorders, several chronic pain syndromes and preeclampsia.

Future studies would be important to address sex-related differences in the protective role of SPM and additional pleiotropic effects of these mediators. It would also be useful to expand the knowledge on resolution of inflammation to other diseases of the neuroscience and cardiometabolic areas, such as depression, heart failure and diabetes, given the growing body of evidence supporting the efficacy of SPM precursors in the improvement of these conditions (Liao et al., 2019; Delpino et al., 2022; Djoussé et al., 2022).
